# Retraction: Antiulcer secondary metabolites from *Elaeocarpus grandis*, family Elaeocarpaceae, supported by *in silico* studies

**DOI:** 10.1039/d5ra90005k

**Published:** 2025-01-13

**Authors:** Radwa Taher Mohie El-Dien, Sherif A. Maher, Usama Ramadan Abdelmohsen, Asmaa M. AboulMagd, Mostafa Ahmed Fouad, Mohamed Salah Kamel

**Affiliations:** a Department of Pharmacognosy, Faculty of Pharmacy, Deraya University, University Zone 61111 New Minia City Egypt; b Department of Biochemistry, Faculty of Pharmacy, Deraya University, University Zone 61111 New Minia City Egypt; c Department of Pharmacognosy, Faculty of Pharmacy, Minia University 61519 Minia Egypt usama.ramadan@mu.edu.eg; d Department of Pharmaceutical Chemistry, Faculty of Pharmacy, Nahda University 62513 Beni Suef Egypt

## Abstract

Retraction of ‘Antiulcer secondary metabolites from *Elaeocarpus grandis*, family Elaeocarpaceae, supported by *in silico* studies’ by Radwa Taher Mohie El-Dien *et al.*, *RSC Adv.*, 2020, **10**, 34788–34799, https://doi.org/10.1039/D0RA06104B.

*RSC Advances* hereby wholly retracts this article as there is insufficient data to support the structure of the novel compound reported, namely grandisine H (**1**). In the characterisation data provided, the H NMR data shows inconsistencies with what would be expected for this compound. A H–H COSY spectrum has since been obtained to unambiguously determine the structure. However, a H–H COSY correlation between the proposed signals for H-5 (at 4.65 ppm) and H-6 (at 3.37 ppm) would have been expected and this was not obtained. A spin system incorporating H5, H6 and extending to H7, 8 and 9 would have also been expected, and this was not found to be the case. The H–H COSY characterisation data was therefore not able to unambiguously determine the structure of the compound, which in consideration with inconsistencies in the H NMR data, casts doubt on the determination of this novel compound.

Removing the reference to the novel compound would remove much of the originality of the article, and therefore the conclusions are not fully supported.

*RSC Advances* apologises for any inconvenience to readers.

All authors were informed of the retraction of this article. Usama Ramadan Abdelmohsen, Asmaa M. AboulMagd and Radwa Taher Mohie El-Dien have signed the notice. Sherif A. Maher, Mostafa Ahmed Fouad and Mohamed Salah Kamel have not responded to say that they will sign the notice.

Date: 8th January 2025

Retraction endorsed by Laura Fisher, Executive Editor, *RSC Advances*

